# Motivational profiles and their relationship with responsibility, school social climate and resilience in high school students

**DOI:** 10.1371/journal.pone.0256293

**Published:** 2021-08-25

**Authors:** David Manzano-Sánchez, Alberto Gómez-Marmol, José Francisco Jiménez-Parra, Isabel Gil Bohórquez, Alfonso Valero-Valenzuela

**Affiliations:** 1 Department of Physical Activity and Sport, CEI Campus Mare Nostrum, University of Murcia, Santiago de la Ribera, Murcia, Spain; 2 Department of Plastic, Music and Dynamic Expression, Faculty of Education, University of Murcia, Murcia, Spain; 3 E- Learning Facilitator, Hasting, United Kingdom; Aalborg University, DENMARK

## Abstract

The aim of this study was to assess the relationships among motivational profiles, their responsibility levels, the school social climate and resilience, and the differences according to gender and age of students from different secondary schools in Spain. A sample of 768 students (mean age of 13.84 years), 314 boys (46.1%) and 354 girls (53.9%) was used. The measurements taken concerned: personal and social responsibility, basic psychological need satisfaction, motivation, resilience and school social climate. Bivariate correlation, cluster and multivariate analyses were carried out. The cluster analysis was made using the Motivation toward Education Scale with its different variables (intrinsic, identified, introjected, external motivation and amotivation), revealing four profiles: low quality (1, low values in all motivational variables except in amotivation), low quantity (2, low values), high quantity (3, high values), and high quality (4, high values except in amotivation). The contrast in comparisons shows differences in resilience, personal and social responsibility, teacher climate and school climate (p < .001). The group with the highest values in resilience, basic psychological needs, responsibility and school social climate was that with a high quality profile. There were statistical differences in all variables with respect to the low quantity and low quality groups (p < .001), while the high quantity group showed statistical differences only in personal and social responsibility (p < .001). The low quality group had the lowest values among all the variables, with statistical differences with respect to all groups (p < .001). On the other hand, there were more boys than girls associated with high quantity, without differences in their age. In conclusion, high quality motivation profiles (those with high or low amotivation values and high values in autonomous and controlling motivation), also have a higher satisfaction of basic psychological needs. Moreover, these students are more resilient, show more responsibility and enhance the school/teaching social climate, while low quality and /or quantity motivation, influence negatively on these variables.

## Introduction

In the educational field, motivation is considered a fundamental element that has a decisive influence on the academic performance and well-being of students [1, 2], as well as their self-confidence and self-improvement [3]. These variables, along with responsibility, play a key role in the satisfaction of basic needs [4] and in the creation of an adequate classroom climate [5], to enhance the integral development of students and the teaching-learning processes.

To understand motivational processes there is a theoretical construct, the "Self-Determination Theory" (SDT) [6], which forms the basis of the present study, referring as it does to the development and functioning of human motivation and personality in certain social contexts, distinguishing between an autonomous motivation and a more controlling one [7]. Therefore, it forms a broad framework that facilitates the understanding of intrinsic motivation, extrinsic motivation and psychological well-being, issues of direct importance for educational centres [8]. Following the SDT, Ryan and Deci [6] establish that people have three basic psychological needs that are essential and inherent (autonomy, competence and relatedness) which promote optimal functioning [9], but whose frustration can lead to maladjustment [10].

Within the framework of the SDT, Vallerand’s [11] hierarchical model of motivation contains several postulates among which a series of social factors or antecedents are indicated, such as responsibility [4], which can induce the satisfaction of basic psychological needs. This, in turn, would lead to an improvement in a more self-determined motivation, such as an enhancement of the classroom climate, in addition to different behavioral, cognitive and affective consequences that are more adaptive [12, 13], and among which the concept of resilience can be found.

Following this line, recent studies suggest establishing a direct relationship between the personal and social responsibility of adolescents and an improvement of the classroom climate [14]. This variable depends on various socio-emotional factors and self-efficacy, forming a complex ecological framework [15] that stimulates learning by improving student motivation, basic psychological needs and academic performance [16, 17].

Within the educational context, students are continuously subjected to adverse circumstances that lead to potentially stressful situations, which each adolescent must deal with Trigueros et al. [18]. One of the tools for facing adversities in the field of pedagogy is resilience [19]. Research such as that of Fletcher and Sarkar [20] and León-Guereño, Tapia-Serrano and Sánchez-Miguel [21] in the context of sports, show the positive relationship that exists between the most self-determined motivation and the personal growth of athletes, since the optimal development of the level of resilience is due to a series of psychological factors among which is a high internal motivation [22]. However, there are practically no studies that have related these variables in the educational field, since the scientific literature has focused on analyzing the motivational processes of students in decision making, without taking into account the influence of emotions and resilience [18, 23]. On the other hand, the implementation of active methodologies in the classroom, based on motivation, show their effectiveness in improving resilience [24].

Although the SDT does not go into detail about possible differences at gander or age level, there are numerous scientific investigations that have studied academic motivation based on gender [25–28], mostly finding that males show higher values in self-determined motivation than females. The studies by Granero-Gallegos et al. [27] and Granero-Gallegos and Gómez-López [28] present boys as more related with the "high motivation" profile, while the "low motivation" profile is mostly associated with girls. However, some studies like Ardenska et al. [25] show that girls had higher internal motivation and less a motivaton than boys.

With regard to age and school course, different studies [29] reported that older students have a higher quality profile. On the other hand, the study of Ntoumanis, Barkoukis and Thøgersen-Ntoumanis [30] shows that younger students had higher quality and quantity of motivation.

Taking into consideration the above and under the SDT paradigm, the main objectives of this study are the following: (1) to analyze the motivational profiles of secondary school students following the theory of self-determination, (2) to analyze how different motivational profiles relate to levels of responsibility, school social climate and resilience, and (3) to analyze differences in motivational profiles according to the gender and age of the students.

## Materials and methods

### Study design and participants

A transversal and quantitative study was carried out and informed consents (confidential data treatment, participation in the study) were requested from the students and their parents.

Participants were selected from four different public secondary schools based on accessibility and convenience. The sample of students originally consisted of 893 participants. The following exclusion criteria were established: (a) to complete all test scales and (b) to complete at least 90% of the test items (excluding double answers). After applying the exclusion criteria and calculating Mahalanobis distance to remove outliers and atypical values, the final sample consisted of 768 participants with ages from 11 to 18 years old (M = 13.84 SD = 1.35), of which 314 were boys (46.1%) and 354 girls (53.9%).

### Procedure

Before completing the questionnaire, the main researchers contacted the different centres. After that, the participants were given an information sheet and were asked to sign an informed consent form. The students answered a questionnaire during a session in a quiet environment lasting 35 minutes. First, students watched a power point presentation about how to complete the questionnaire, after that the teacher read the questions in order to ensure they were understood. A teacher and one of the researchers were with them all the time to solve any possible doubts. The participants were requested to provide true answers. Participants were informed of the purpose of the research and were told that it was voluntary and confidential.

### Instruments

A closed-question questionnaire was used in the present study, it had two parts, the first one consisted of socio-demographic variables, and the second part contained the scales used in the study.

1. Personal and Social Responsibility Questionnaire (PSRQ): to measure personal and social responsibility levels. It was adapted to the school context by Li et al. [31] and for Spanish by Escartí et al. [32] and validated in a sample of 9 to 15 years old students. This scale consists of 14 items, seven to assess social responsibility (e.g., “I help others”) and seven for personal responsibility (e.g., “I set goals”). The answers were provided on a Likert-type scale ranging from 1 (totally disagree) to 6 (totally agree). Reliability in the pre-test was α = .846 for social responsibility and α = .736 for personal responsibility.
2. Psychological Need Satisfaction in Exercise (PNSE): to measure the satisfaction of the need of social competence, autonomy and relationships. The scale adapted for Spanish and to the education context by Moreno et al. [33] and validated in a sample of 12–16 years old. This scale consists of 18 items, six to evaluate each need: competence (e.g., “I am confident to perform the most challenging tasks”), autonomy (e.g., “I believe I can make decisions during my classes”) and relationships with others (e.g., “I feel attached to my class mates because they accept me as I am”). These were preceded by the sentence “During my class…” and the answers were provided on a Likert-type scale ranging from 1 (False) to 5 (True). Reliability in the pre-test was α = .789 for autonomy, α = .787 for competence and α = .797 for relationships. Moreover, the psychological mediator index (PMI) was applied to evaluate the three variables jointly, yielding an internal consistency of α = .880.
3. Motivation toward Education Scale (in French, EME): to measure motivation from the most self-determined types to the most external causes and amotivation. The Spanish version of the Échelle de Motivation en Éducation [34] validated by Nuñez, Martín-Albo and Navarro [35] was used. The questionnaire passed a reliability test in order to check the understanding of the student sample in the same way as the others. The questionnaire consisted of seven subscales, called intrinsic motivation, to reveal; information (e.g., “because I feel pleasure and satisfaction when I learn new things”), accomplishment (e.g., “for the pleasure I feel when I improve my academic performance”), the experience of sensations (e.g., “because reading about topics I find interesting stimulates me”), identification of regulation (e.g., “because it will allow me to access the job market in my preferred field”), introjected motivation (e.g., “to prove to myself that I am an intelligent person”), external motivation (e.g., “to get a more prestigious job”) and amotivation (e.g., “I don’t know, I don’t understand what I’m doing at high school”). The instrument was composed of 28 items preceded by the sentence “I go to school / high school because…”, with a seven-point Likert-type scale, from 1 (totally disagree) to 7 (totally agree) and distributed into seven subscales, five of them containing four items and two of them containing three items. We used the formula from controlling motivation (external regulation + introjected regulation, α = .922), autonomous motivation (identified regulation + intrinsic motivation, α = .830) and amotivation (α = .809) as recommended by other authors [29]. The Self-Determination Theory (SDT) says that autonomous motivation and the satisfaction of basic psychological needs are the most adaptative profiles. For this reason, it can be said that “high quality” refers to a more adaptative motivation (specially, internal motivation) than external and amotivation. The autonomous motivation is denominated like “high quality” and amotivation like “low quality” [29].
4. Resilience scale (RS-14) [36] was used to measure the degree of individual resilience, considered as a positive personality characteristic that allows the individual to adapt to adverse situations. The RS-14 measures two factors: Factor I: Personal Competence (11 items, self-confidence, independence, decision, resourcefulness and perseverance; e.g., “My life makes sense”) and Factor II: Acceptance of oneself and of life (3 items, adaptability, balance, flexibility and an established life perspective; e.g., “I am a disciplined person”). A six-point Likert-type scale was used, ranging from 1 (totally disagree) to 6 (totally agree). We used the whole scale and the value of reliability was α = .876.
5. A questionnaire to assess school social climate (CECSCE): to evaluate the climate perceived by students with regard to their class, teacher and school. It was designed by Trianes et al. [37], and validated in a 12–14 years old student sample. The questionnaire consists of two subscales called “School climate” (e.g., “Students are really willing to learn”), made up of eight items, and “Teaching climate” (e.g., “Teachers of this school are friendly to students”), composed of six items. A five-point Likert-type scale was used, ranging from 1 (totally disagree) to 5 (totally agree). The internal consistency analysis yielded a value of α = .785 for school climate and α = .834 for teaching climate.

### Data analysis

First, descriptive statistics (i.e., mean, SD, skewness, and kurtosis) were calculated for the variables considered. Skewness and kurtosis were used to check the normality of data (values < 1.96 are considered as normal) [38]. Cronbach’s alpha coefficient was estimated to examine the reliability of each variable analyzed, which is acceptable with values over .70 [39]. The second step was to check the correlation between the variables, where our findings supported the study of Hair et al. [40] with values < .80 indicating the absence of multicollinearity between the variables.

Third, we checked the student’s profiles using a two-step cluster analysis approach using a combination of hierarchical and non-hierarchical methods [40]. Subsequently, a hierarchical conglomerate analysis was performed using Ward’s model, standardizing the variables and using the Z scores of autonomous motivation and controlled motivation. We selected the most suitable solution, checking the squared Euclidean distance and analyzing the dendrogram. Furthermore, a univariate analysis of variance was performed to inspect the explanatory power of the cluster solution for each of the constituting clustering variables. In the second step, a nonhierarchical cluster analysis (k means) was carried out, using the initial cluster centres that emerged from the hierarchical cluster analysis. In addition, we carried out a double-split cross-validation approach to inspect its stability. For this, the sample was randomly split into halves, and the procedure was again applied to each subsample. Both solutions were averaged to judge the degree of agreement in relation to the original cluster solution, using the Cohen’s kappa (κ) index with a value of.74, indicating a suitable value above.60, following Breckenridge [41].

Finally, in order to check whether there were significant differences in the profiles regarding resilience and responsibility, a multivariate analysis of variance (MANOVA) was performed, calculating the main effect. In those cases in which a significant statistical difference was found, a post hoc contrast of comparisons test was carried out, using the Bonferroni correction to determine between which clusters there would be statistically significant differences. In addition, the effect size was calculated in terms of partial eta squared (η2), considering a small effect size with values < .01, medium effect between.01 and.06, and large effect with value >.14 [42]. Prior to this analysis, Box’s test was employed to analyze the homogeneity of covariances [38]. All analysis was performed with IBM SPSS, v. 23.0 establishing the level of significance p < .05.

### Ethics statement

Insofar as ethical rules are concerned, the study previously received the approval of the Ethics Committee of the University of Murcia (1685/2017). All participants were treated in agreement with the ethical guidelines regarding consent, confidentiality and anonymity of the answers. In addition, a written informed consent was made by students, their parents and the directors of the schools.

## Results

### Descriptive statistic, reliability and correlations

Table 1 presents the mean values, standard deviation, asymmetry and kurtosis values, reliability of the variables and the correlations. The values of normality and reliability were considered acceptable (*α* >.70). There are correlations between all variables except between those of amotivation and school climate, and amotivation and autonomy. None of the variables had a value above 0.80, except PMI, because it was an index (autonomy + competence + relatedness)/3. All correlations were positive except for the Amotivation variable.

**Table 1:** Descriptive analysis and correlations.

		*R*	*M*	*SD*	*A*	*K*	*α*	2	3	4	5	6	7	8	9	10	11	12
1	Controlling Motivation	1–7	5.52	1.08	-0.83	0.36	.922	.714**	-.146**	.513**	.544**	.411**	.583**	.626**	.561**	.456**	.444**	.368**
2	Autonomous Motivation	1–7	5.10	1.11	-0.63	0.13	.830		-.240**	.580**	.604**	.449**	.649**	.642**	.592**	.513**	.524**	.455**
3	Amotivation	1–7	2.22	1.41	1.14	0.56	.809			0.000	-.138**	-.121**	-.101**	-.115**	-.214**	-.175**	-.153**	-0.042
4	Autonomy	1–5	3.37	0.90	-0.35	-0.25	.789				.660**	.476**	.853**	.539**	.453**	.413**	.594**	.550**
5	Competence	1–5	3.75	0.83	-0.71	0.42	.787					.522**	.861**	.659**	.553**	.470**	.599**	.507**
6	Relatedness	1–5	3.96	0.89	-0.93	0.40	.797						.799**	.515**	.395**	.462**	.518**	.547**
7	PBN	1–5	3.69	0.73	-0.60	0.36	.880							.679**	.556**	.534**	.681**	.639**
8	Resilience	1–6	5.24	1.00	-0.67	0.16	.876								.535**	.497**	.489**	.460**
9	Personal responsibility	1–6	4.72	0.85	-0.81	0.39	.736									.645**	.454**	.359**
10	Social responsibility	1–6	4.93	0.81	-0.89	0.41	.846										.472**	.454**
11	Teaching climate	1–5	3.66	0.82	-0.45	-0.35	.785											.707**
12	School climate	1–5	3.43	0.79	-0.31	-0.23	.834											

R = Range M = Mean, ST = Standard Deviation; A = Asymmetry; K = Kurtosis; BPN = Basic Psychological Needs;

* = p < .05;

**p < .001.

### Cluster analysis to obtain motivational profiles

After removing outlies (Z > +-3 and Mahalanobis distance at p < .001) we started with the first step using hierarchical cluster analysis. The dendrogram and the agglomeration coefficients reflected that there were three possible solutions which were seven, four, three, and six profiles with a movement of 14.63%, 17.01%, 24.8% and 37.51%. The final selection of the cluster was made with three criteria. First, due to the fact that the coefficient increased highly with the movement between four and three profiles (7.37%) and didn’t change substantially between four and seven (2.38%), the implication was that the four-cluster solution could generate a higher level of heterogeneity than three or six. Secondly, we followed other research that supports the realization of these profiles. In this way, based on the results and taking into account the main theoretical contributions made in this regard [29, 38], the structure formed by 4 clusters was chosen as the most convenient solution. Finally, we checked that the four-cluster solution was the profile that explained the variance of the clustering (autonomous motivation, controlling motivation, amotivation and PMI) with an explained variance for the set of variables of 70.4% (R^2^ = .704; R = .839). On the other hand, the cluster had significant correlations in p < .001 for amotivation, autonomous and controlling motivation and PMI.

**Fig 1:**
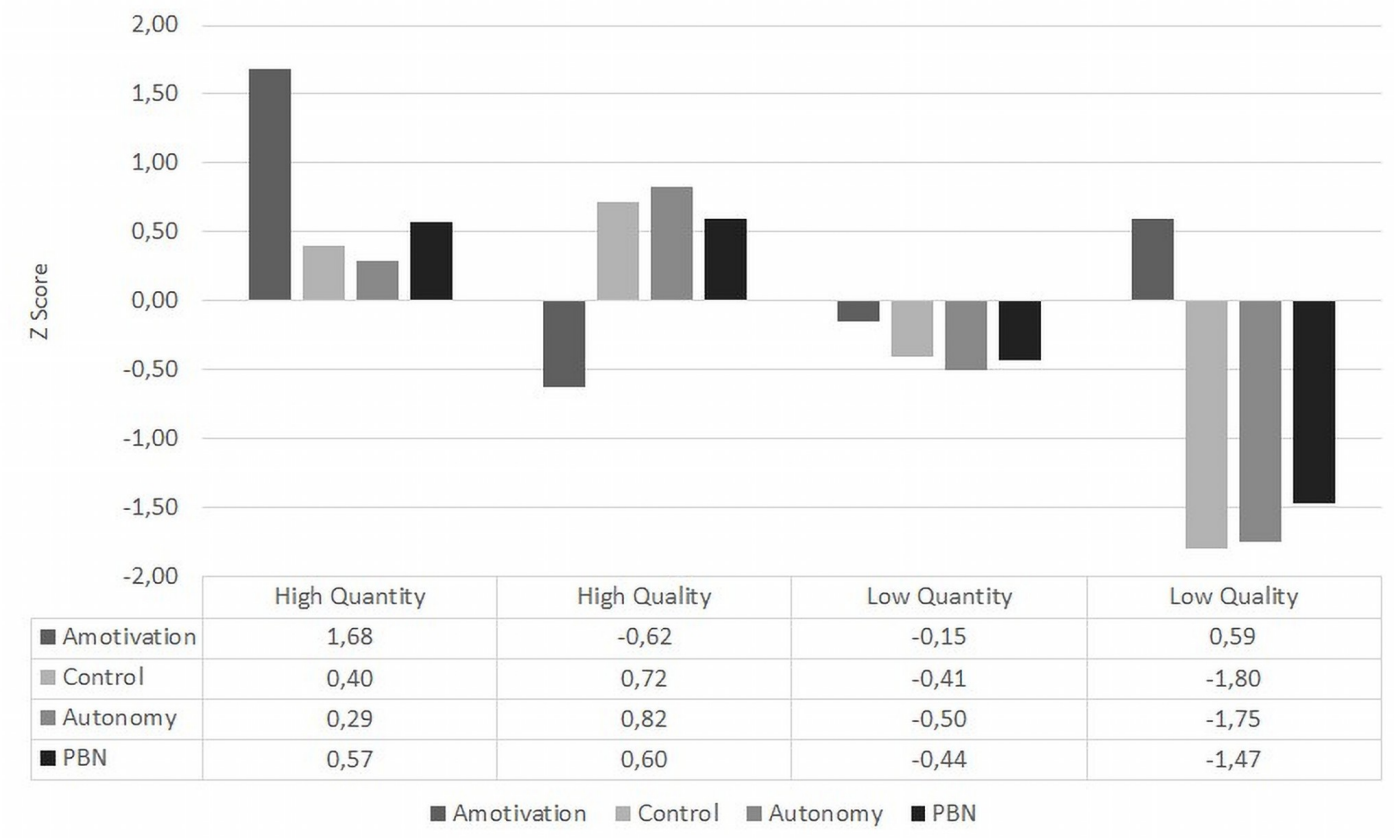
Motivational profiles in students.

The nonhierarchical cluster confirmed the four-cluster solution (Fig 1 and Table 2). The first profile was denominated “high quantity” (n = 103, 13.4%) with high values in all variables, the second profile “high quality” (n = 310, 40.4%) had high levels in autonomous, controlling motivation and BPN and low levels of amotivation, the third profile (n = 269, 35%) was denominated “low quantity” with low values in all variables. Finally, the fourth profile “low quality” (n = 86, 11.2%) with low values in autonomous and controlling motivation and BPN and high amotivation levels. On the other hand, Table 2 shows the differences among the variables that configured the cluster solution. They had a multivariate effect (Box’s value = 367.383, f = 12.083, p = < .001), pointing to the violation of the assumption of homogeneity of covariances and suggesting the use of Pillai’s trace as a test statistic [38] showing a Pillai’s trace value of 1.407 (f = 168.471).

**Table 2:** Differences in variables from motivational profiles.

	High Quantity		High Quality		Low Quantity		Low Quality				
	*M*	SD	*M*	SD	*M*	SD	*M*	SD	*F*	*p*	*eTa*
Amotivation	1.68	0.73	-0.62	0.36	-0.15	0.67	0.59	0.92	424.20	< .001**	.625
Controlling Motivation	0.40	0.64	0.72	0.47	-0.41	0.63	-1.80	0.75	483.52	< .001**	.655
Autonomous Motivation	0.29	0.63	0.82	0.45	-0.50	0.52	-1.75	0.71	640.99	< .001**	.716
PBN	0.57	0.64	0.60	0.69	-0.44	0.69	-1.47	0.92	250.13	< .001**	.496
Box’s = 367.383 (f = 12.083) p = < .001											
Pillai’s trace = 1.407 (f = 168.471) p = < .001											

M = Mean, SD = Standard Deviation; PBN = Basic Psychological Needs; eTa = Partial eta squared.

* = p < .05;

** = p < .001.

### Difference analysis between clusters

The multivariate analysis of variance test (Table 3) showed a statistically significant multivariate effect for the four motivational profiles, M de box = 151.076 (F = 3.303, p = < .001), Pillai’s trace = .579, F = 36.451, p = < .001). The four motivational profiles identified significantly differed from one another with respect to personal and social responsibility, resilience, teacher climate and centre climate. The results (Table 4), using multiple comparisons, contrast with Bonferroni’s correction.

**Table 3:** Differences in resilience, responsibility and climate from motivational profiles.

	High Quantity		High Quality		Low Quantity		Low Quality				
	M	SD	M	SD	M	SD	M	SD	F	p	eTa
Resilience	5.61	0.67	5.80	0.68	4.90	0.84	3.81	0.92	180.061	< .001**	0.414
Personal responsibility	4.80	0.61	5.20	0.58	4.51	0.76	3.55	0.84	144.051	< .001**	0.361
Social responsibility	4.97	0.68	5.32	0.61	4.75	0.73	4.02	0.91	87.521	< .001**	0.256
Teaching climate	3.89	0.71	4.09	0.65	3.36	0.70	2.80	0.77	105.522	< .001**	0.293
School climate	3.74	0.78	3.78	0.65	3.14	0.73	2.74	0.59	77.117	< .001**	0.232
Box’s = 151.076 (F = 3.303) p = < .001											
Pillai’s trace = .579 (F = 36.451) p = < .001											

M = Mean, SD = Standard Deviation; eTa = Partial eta squared.

* = p < .05;

** = p < .001.

**Table 4:** Multiple comparisons contrast.

	1 vs. 2	1 vs.3	1 vs. 4	2 vs. 3	2 vs. 4	3 vs. 4
Resilience	-0.18	.71**	1.80**	. 90**	1.98**	1.09**
Personal responsibility	-.40**	.30**	1.25**	.69**	1.65**	.73**
Social responsibility	-.35**	.22*	.95**	.57**	1.30**	.55**
Teaching climate	-0.20	.54**	1.09**	.73**	1.29**	.55**
School climate	-0.04	.60**	1.00**	.64**	1.04**	.39**

1 = high quantity, 2 high quality, 3 = low quantity, 4 = low quality,

* = p < .05;

** = p < .001.

Post hoc analysis reported that, in the case of personal and social responsibility, significant differences were found between the 4 motivational profiles, with the higher values being for the high quality profile, followed by high quantity. In third position appears low quantity and finally the low quality profile. Furthermore, when considering resilience and teacher or centre climate, significant differences were found among the 4 motivational profiles except, that is, for the comparison between the high quantity and high quantity profiles, where none of the differences reached significant values.

### Differences according to gender and age

In order to check the differences in the distribution of the motivational profiles found in terms of gender and the course, it was decided to perform a difference analysis using Pearson’s chi-square statistic. This goodness-of-fit test compares the observed and expected frequencies in each category to test whether all categories contain the same proportion of values or whether each category contains a user-specified proportion of values. Likewise, the use of the corrected typified residuals provides us with information on where these differences are found, since residuals equal to or greater than 1.90 are considered as indicators that show dependence between these 2 categories and that, therefore, the differences are significant.

In terms of gender, Table 5 reports that there were more boys than girls associated with high quantity. However, student age did not show any marked statistical differences in any of the categories.

**Table 5:** Differences according to gender and age.

	High Quantity			High Quality			Low Quantity			Low Quality					
	Total	%	R	Total	%	R	Total	%	R	Total	%	R	X^2^	gl	p
Men	69	67.0%	2.9	154	49.7%	-1.9	142	52.8%	-.5	49	57.0%	.6	9.789	3	0.02*
Women	34	33.0%	-2.9	156	50.3%	1.9	127	47.2%	.5	37	43.0%	-.6			
11–13	44	42.7%	.3	131	42.3%	.4	113	42.0%	.2	30	34.9%	-1.3	3.944	6	.684
14–15	50	48.5%	.4	145	46.8%	.0	119	44.2%	-1.0	45	52.3%	1.1			
16–18	9	8.7%	-1.0	34	11.0%	-.6	37	13.8%	1.2	11	12.8%	.3			

R = Standardized Residual, SD = Standard Deviation; PBN = Basic Psychological Needs; x^2^ = chi squared.

## Discussion

The main objectives of this study were to analyze the motivational profiles of a sample of secondary school students following the SDT, how they relate to responsibility levels, the school social climate and resilience, and their possible differences according to the students’ gender and age. In broad terms, the results confirm the conclusions reported by Sánchez-Oliva et al. [29] since they also found four motivation profiles among 1690 secondary school students (12–16 years old), showing that the profiles with higher levels of motivation had more adaptive consequences. In our study, the profiles were similar, but it is important to highlight that one of the profiles had a higher motivation and amotivation level (the same as Sánchez-Oliva et al. [29]) and this profile could be following a “response pattern”). However, it is not in line with the studies by Haerens et al. [43] and Yli-Piipari et al. [44], which may be due to the use of different variables or instruments to build these profiles or, even, samples with different characteristics (e.g. primary school students or professional sports people).

Moreover, correlation analyses among the studied variables are consistent with the profiles’ characteristics and also, with prior research. In this sense, Li et al. [31] found, in a sample of 253 middle school students, a positive correlation between motivation and responsibility. In that correlation, Menéndez and Fernández-Río [45] also added basic psychological needs. With regard to school climate, in their research for validating a questionnaire with more than 800 secondary school students, Fernández-Río et al. [46] affirmed that those contexts with a good school social climate tend to be made up of students with high levels of responsibility. Finally, regarding the relationship with resilience, Soetanto, Mullins and Achour [47] remarked that resilience is a variable that has often been analyzed by other disciplines like business or economics. In the education field, to the best of our knowledge, this is the first research that attempts to analyze whether they are related and how. There is, however, some other research whose results may contribute to this analysis. For instance, nurses’ engagement when experiencing end-of-life care work, is mediated by, among others, resilience and responsibility [48]. Another example can be found in the results obtained by Hebbani and Srinivasan [49]. They affirmed that assuming more social responsibilities in each family influences the development of resilience. It should, however, be pointed out that neither of these studies was carried out in the context of the school.

With regard to gender and age, our results do not confirm other research conclusions. This investigation did not find differences based on age but it did regarding gender. The greater proportion of boys in the high quantity profile is in line with the findings of Sánchez-Oliva et al. [29] and Granero-Gallegos et al. [50]. However, while they [29] reported that those students in higher courses tended to be grouped in the high quality profile, the present research did not report any significant difference. In this sense, Ntoumanis, Barkoukis and Thøgersen-Ntoumanis [30], found good levels in both the amount and direction (quantity and quality) in younger students in comparison to older students.

### Strengths and limitations

This study presents some limitations, mainly due to its cross-sectional and descriptive work, where causality relationships cannot be established. Furthermore, it should be taken into consideration that the psychological need satisfaction instrument was created for the context of the exercise, and there could be some bias when it is used in a school context. On the other hand, the presence of the teacher could have influenced some student’s answers. Future investigations may want to contemplate the possibility of carrying out a prediction analysis, to verify whether the relationships of this study can follow Vallerand’s [11] hierarchical model, considering responsibility as a trigger variable for motivation and basic psychological needs, and whether, in turn, this leads to such things as resilience and school social climate. In addition, it would be interesting to analyse the existing differences based on socio-demographic variables (gender and age). In line with previous studies, it would be useful to implement experimental studies to develop training programs for teachers giving methodological and motivational strategies for supporting students’ basic psychological needs satisfaction, which would contribute to the creation of a better social climate and more resilience in students’ behaviour. Finally, it could be a good idea to make studies of prediction models to show if the SDT could help to understand some behaviour in students such as their resilience, improving the school climate or their responsibility.

## Conclusions

The students that have greater satisfaction of their basic psychological needs and who feel more motivated are the students with higher resilience and responsibility, in addition to feeling that the school and teaching climate is better. On the other hand, low motivation levels (in quantity or quality) suppose low resilience, personal and social responsibility and school and teaching climate. A high quantity of motivation and satisfaction of psychological needs, is a good way to improve resilience, responsibility and school or teaching climate, but, it is better if amotivation values are low (high quality profile). Finally, there are more men than women in the high quantity profile, but not in the rest of the profiles. We have discovered no differences with regard to the age.
